# When the Body Hardens and the Mind Fragments: Psychosis in Systemic Sclerosis

**DOI:** 10.7759/cureus.104948

**Published:** 2026-03-09

**Authors:** Gonzalo Andrés Pacheco Ortega, César David Tamayo de León, Cristian Alejandro Molina Martínez, Raúl Anwar Garcia-Santos

**Affiliations:** 1 Neurology, Instituto Nacional de Neurología y Neurocirugía Manuel Velasco Suárez, Mexico City, MEX; 2 Neuroradiology, Instituto Nacional de Neurología y Neurocirugía Manuel Velasco Suárez, Mexico City, MEX

**Keywords:** autoimmune disease, autoimmune psychosis, connective tissue disease, psychosis, systemic sclerosis

## Abstract

Systemic sclerosis is a chronic autoimmune disease primarily characterized by fibrosis and vasculopathy; neurological involvement is considered uncommon. We present the case of a 27-year-old woman with a history of asthma, Raynaud’s phenomenon, and chronic facial dermatosis, who developed an acute psychotic syndrome characterized by hallucinations, disorganized speech, and persecutory delusions, with no prior psychiatric history. Physical examination revealed mucocutaneous findings consistent with systemic sclerosis, and neurological evaluation showed altered cognition and behavioral disturbances. Laboratory and cerebrospinal fluid analyses were unremarkable, including negative anti-NMDA receptor antibodies. Transcranial Doppler suggested increased cerebral vascular resistance, raising suspicion of CNS vasculitis. The patient fulfilled the 2013 American College of Rheumatology (ACR)/European League Against Rheumatism (EULAR) classification criteria for systemic sclerosis despite negative specific autoantibodies. Neuropsychiatric symptoms persisted despite antipsychotic therapy, but improved markedly following high-dose intravenous methylprednisolone and cyclophosphamide. This case illustrates a rare and atypical neuropsychiatric manifestation of systemic sclerosis, highlighting the importance of recognizing autoimmune contributions to acute psychosis. Timely immunosuppressive treatment can lead to favorable outcomes even in the absence of definitive serological markers. Further studies are needed to elucidate the pathophysiology of central nervous system involvement in systemic sclerosis and to guide diagnostic and therapeutic strategies for such presentations.

## Introduction

Systemic sclerosis is a complex rheumatologic disease characterized by skin and internal organ fibrosis, as well as vasculopathy [1]. Neurological involvement in systemic sclerosis has traditionally been considered rare and secondary to systemic pathology; however, recent evidence suggests the possibility of a primary process affecting the nervous system, as described by Prajjwal et al. [2]. Although the pathophysiology remains unclear, it has been postulated that systemic sclerosis may induce primary vascular changes, including calcification of arterioles and capillaries within the brain parenchyma. Endothelial damage, precipitated by autoantibodies and reactive oxygen species, may result in luminal narrowing, obliteration of blood vessels, and intimal hyperplasia [2].

The clinical manifestations of neurological involvement in systemic sclerosis are diverse and may include epileptic seizures, headaches, cranial nerve deficits, cognitive alterations, transverse myelitis, myopathy, and psychiatric disturbances such as depression, dysthymia, suicidal ideation, psychotic syndromes, and paranoid thoughts. A systematic review evaluating neurological involvement in patients with systemic sclerosis also assessed the prevalence of psychiatric manifestations. Among 1,490 patients identified with psychiatric symptoms, depression was the most common (73%), followed by anxiety (23%) and dysthymia (2%). Less common findings included suicidal ideation (0.4%), psychotic syndromes (0.13%), and paranoid thoughts (0.13%) [3].

In this context, we report the case of a patient with systemic sclerosis who developed an acute psychotic syndrome, representing an atypical manifestation that warrants further analysis and discussion.

## Case presentation

A 27-year-old woman with a history of asthma, Raynaud’s phenomenon, and chronic facial dermatosis presented to the emergency department with an abrupt onset of psychotic symptoms, including visual and auditory hallucinations, disorganized speech, urinary incontinence, emotional lability, and persecutory delusions. The symptoms developed acutely after receiving a message about a potential job interview, in the context of a recent upper respiratory tract infection and ongoing psychosocial stress.

**Figure 1:**
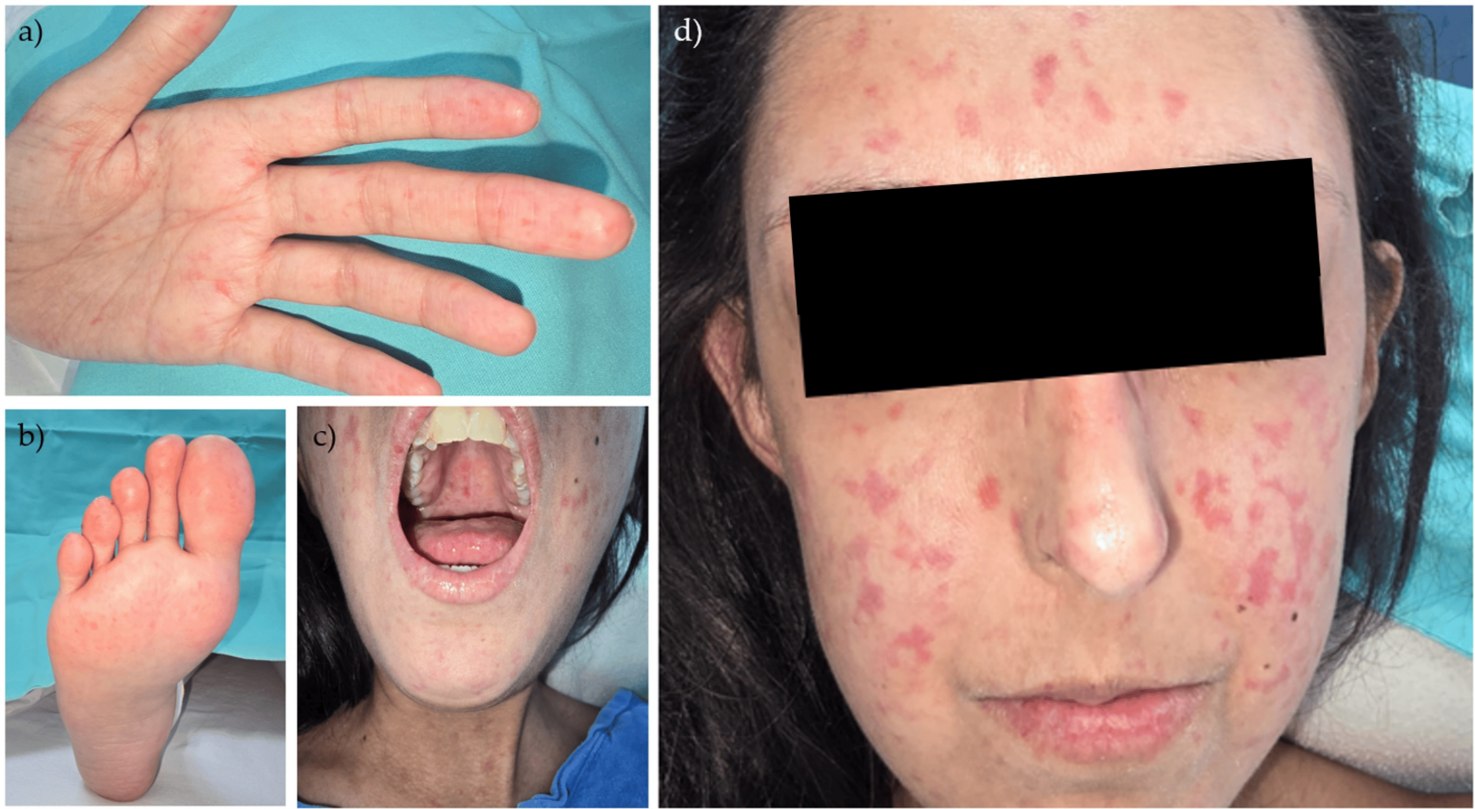
Dermatologic findings (a-b) Sclerodactyly was noted, with shiny, indurated skin over the fingers and reduced skin mobility; (c) Macules on the palate surface; (d) Dermatosis that affects the entire face bilaterally with a tendency toward symmetry. The dermatosis has a monomorphic appearance and is composed of numerous erythematous patches of varying sizes and shapes, with irregular but well-defined borders. Additionally, there is nasal thinning and microstomia.

On physical examination, the patient appeared underweight and alert, though disoriented. Dermatologic assessment revealed multiple non-confluent, erythematous-to-violaceous macules and patches over the face, perioral area, forehead, and neck, with prominent involvement of the malar region. Similar lesions were noted on the palms, soles, and oral mucosa, particularly on the hard palate and buccal surfaces (Figure 1). The skin over the digits appeared tight and mildly hypopigmented, with reduced pliability, and acral areas were cool to the touch. Neurological examination revealed fluctuating attention, a suspicious demeanor, psychomotor agitation, and paranoid ideation. Cranial nerves were intact; muscle strength was preserved, and reflexes were symmetrical, with bilateral flexor plantar responses.

Initial stabilization involved intramuscular olanzapine and intravenous dexmedetomidine. Neuropsychiatric evaluation described a red-flag psychotic syndrome suggestive of autoimmune etiology. There was no prior psychiatric history; however, the patient's mother reported progressive anxiety and emotional distress following her father's death three years earlier.

Cerebrospinal fluid (CSF) analysis showed clear, colorless fluid with a cell count of 2/mm³, glucose of 51 mg/dL, and protein of 37 mg/dL. Anti-NMDA receptor antibodies in CSF were negative. Electroencephalogram showed diffuse slowing of the background activity without epileptiform discharges, consistent with nonspecific cerebral dysfunction [4]. Urinalysis was unremarkable.

**Figure 2:**
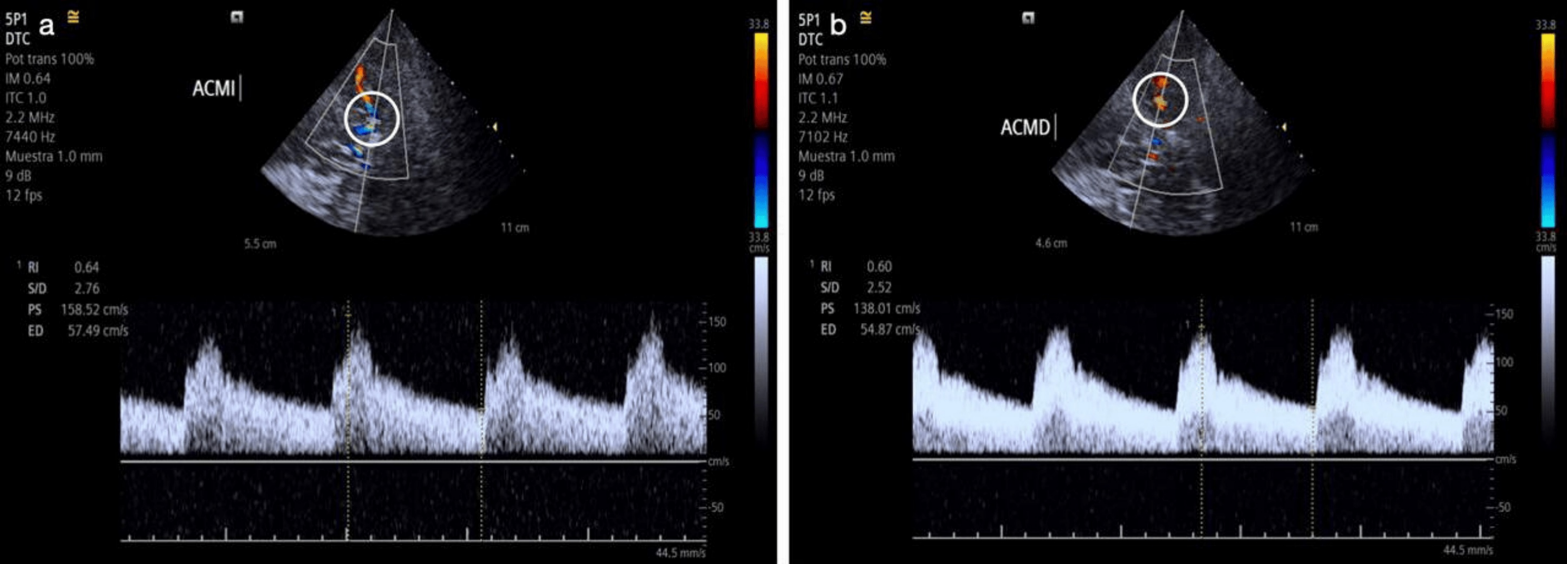
Transcranial Doppler ultrasound Transcranial Doppler ultrasound reveals elevated cerebral blood flow velocity (CBFV) in the left middle cerebral artery (MCA) with peak systolic velocity (PSV) of 158.52 cm/s and a mean velocity (MV) of 91.17 cm/s (a). The right MCA demonstrates mildly increased PSV of 138.01 cm/s, with preserved MV of 82.58 cm/s (b). Open circles indicate the insonated MCA segments.

A transcranial Doppler ultrasound showed elevated systolic velocities in the middle cerebral arteries bilaterally (138/54 cm/s on the right, 158/57 cm/s on the left), with normal findings in the anterior and posterior cerebral arteries (Figure 2).

**Figure 3:**
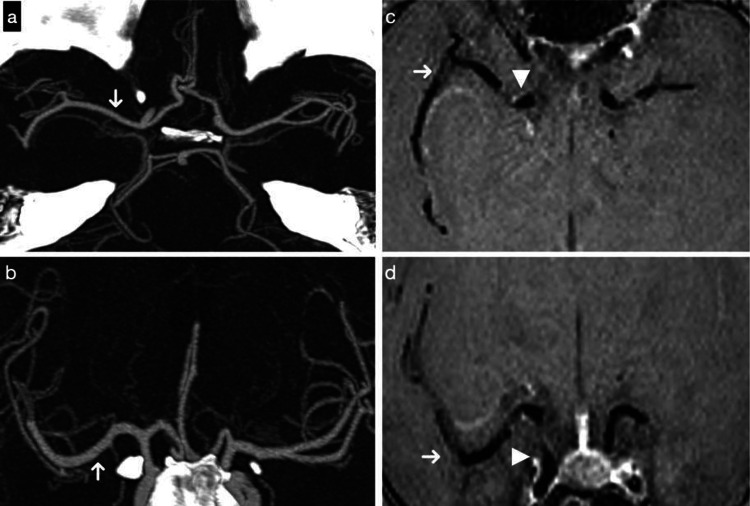
Multimodal evaluation of the circle of Willis and middle cerebral arteries (a-b) Axial and coronal oblique reformatted images from CT angiography show no evidence of luminal narrowing. The arrows depicts M1 segment of the right middle cerebral artery (MCA). (c-d) Axial and coronal oblique contrast-enhanced 3T vessel wall MRI (VWI) demonstrates normal wall thickness and absence of pathological enhancement in the M1 and M2 segments of the right MCA (arrows), as well as in the distal intracranial ICA (arrowheads). VWI: vessel wall imaging; ICA: internal carotid artery

CT angiography and MRI were subsequently performed (Figure 3). Transthoracic echocardiography revealed normal biventricular systolic function, no significant valvular disease, and low probability of pulmonary hypertension.

Based on the constellation of mucocutaneous, neurological, and vascular findings, a systemic autoimmune process with central nervous system involvement was suspected. With support from the rheumatology service, the patient fulfilled the 2013 American College of Rheumatology (ACR)/European League Against Rheumatism (EULAR) classification criteria for systemic sclerosis [5]. Skin thickening of the fingers extending proximal to the metacarpophalangeal joints was present, which alone constitutes a sufficient criterion for classification. Additional supportive features were also identified (Table 1). Subsequent serologic testing revealed negative anti-centromere and anti-Scl-70 antibodies, as well as normal anti-dsDNA and SSA/SSB titers.

**Table 1:** Application of the 2013 ACR/EULAR classification criteria for systemic sclerosis in our patient The 2013 ACR/EULAR classification criteria for systemic sclerosis assign weighted scores to clinical and laboratory features. A total score ≥9 is required for classification. Skin thickening of the fingers extending proximal to the metacarpophalangeal joints constitutes a sufficient criterion for classification. In this patient, this feature was present, and additional clinical findings consistent with systemic sclerosis were also identified. ACR: American College of Rheumatology; EULAR: European League Against Rheumatism

Item	Weight/score
Skin thickening of the fingers of both hands extending proximal to the metacarpophalangeal joints (sufficient criterion)	9 points
Skin thickening of the fingers (sclerodactyly of the fingers)	Present
Telangiectasia	Present
Abnormal nailfold capillaries	Present
Raynaud's phenomenon	Present

The patient received high-dose intravenous methylprednisolone (1 g/day for five days), followed by a single intravenous dose of cyclophosphamide (1 g). She exhibited progressive clinical improvement, including resolution of hallucinations, normalization of cognitive function, and full recovery of orientation. At discharge, she was prescribed a tapering course of oral prednisone and referred for rheumatology and neurology follow-up. At the one-month follow-up, she remained clinically stable, with no recurrence of neuropsychiatric symptoms.

## Discussion

Systemic sclerosis is a chronic autoimmune disease that can affect multiple organ systems, including the lungs, renal, gastrointestinal, and musculoskeletal systems. However, neurological involvement is neither common nor well studied in this disease. In particular, psychiatric manifestations are not considered part of the classic clinical picture, although several studies have shown a higher prevalence of psychiatric comorbidities compared to the general population, including major depression, anxiety disorders, and cognitive symptoms [6-8]. Nevertheless, neuropsychiatric manifestations such as psychotic episodes are rare and poorly documented, posing a significant diagnostic and therapeutic challenge.

Some psychiatric symptoms may be partially explained by psychosocial factors. For instance, anxiety has been associated with dissatisfaction with physical appearance, severity of gastrointestinal symptoms, and prior hospitalizations. Similarly, depression has been linked to disease activity, impaired mobility, and job loss, among other factors. Emerging evidence suggests that neuropsychiatric manifestations may share common molecular mechanisms. Identified mechanisms include an imbalance between reactive oxygen species production and antioxidant defenses, which appears to play a key role in the fibrotic response and disease progression. Additionally, a potential serotonin dysregulation may contribute both to fibrosis progression and neuropsychiatric manifestations [9].

Several clinical features suggested a secondary etiology for the psychotic syndrome. According to the proposed framework for autoimmune psychosis, certain clinical features should raise suspicion of an underlying immune-mediated process, including rapid progression of symptoms, insufficient response to antipsychotic treatment, and the presence of an autoimmune disorder [10]. In this patient, the acute onset of symptoms, the coexistence of visual and auditory hallucinations, and the lack of clinical response to antipsychotic therapy further supported the consideration of an organic neuropsychiatric syndrome in the context of systemic autoimmune disease. The diagnostic workup began with a lumbar puncture, which tested negative for anti-NMDA receptor antibodies. An immunological panel also revealed no antibody positivity. Transcranial Doppler ultrasound revealed increased resistance in the middle cerebral artery, suggestive of central nervous system vasculitis; however, these findings were not confirmed on magnetic resonance imaging (MRI). Antipsychotic treatment was initiated without clinical improvement. Subsequently, based on imaging findings, high-dose methylprednisolone and cyclophosphamide were administered, leading to marked clinical improvement.

This case highlights the need for further investigation into the neuropsychiatric spectrum of systemic sclerosis. In contrast to autoimmune diseases such as systemic lupus erythematosus and Sjögren’s syndrome (where neuropsychiatric involvement is well characterized), the direct impact of systemic sclerosis on the central nervous system remains poorly understood [11]. The present case raises the possibility that disease mechanisms such as endothelial dysfunction, oxidative stress, and serotonin dysregulation may underlie a neuroinflammatory process that manifests as psychosis. The dramatic clinical response to immunosuppressive therapy supports this hypothesis.

Nevertheless, this case has certain limitations. Although a brain MRI was performed, it did not reveal findings consistent with inflammation or vasculitis, which may reflect either a limitation in sensitivity or a predominance of functional vascular dysregulation without overt structural damage. In addition, the autoimmune serological panel was negative, making it difficult to establish a specific immunologic mechanism. The patient also received antipsychotic treatment during the acute phase, which may have contributed partially to clinical improvement; however, the resolution of symptoms occurred only after the initiation of high-dose corticosteroids and cyclophosphamide, suggesting a more substantial effect from immunosuppression. Finally, although the clinical evolution was favorable, a contribution from psychosocial or functional factors cannot be entirely ruled out. Despite these limitations, the atypical presentation and therapeutic response support the consideration of immune-mediated causes in patients with acute psychotic syndromes and underlying systemic autoimmune diseases. This case expands the recognized neuropsychiatric spectrum of systemic sclerosis and underscores the importance of a multidisciplinary approach in similar clinical scenarios.

## Conclusions

This case illustrates an unusual neuropsychiatric manifestation of systemic sclerosis, presenting as an acute psychotic episode in a young adult without prior psychiatric history. Although such presentations are rarely reported, the clinical evolution and favorable response to immunosuppressive therapy support a possible immune-mediated mechanism affecting the central nervous system. Clinicians should maintain a high index of suspicion for autoimmune involvement when encountering atypical psychiatric syndromes, particularly in patients with systemic features suggestive of connective tissue disease. Further studies are needed to explore the neuropsychiatric spectrum of systemic sclerosis and to determine whether early immunomodulatory treatment may improve outcomes in similar cases.
